# Non-small cell lung cancer-small cell lung cancer transformation as mechanism of resistance to tyrosine kinase inhibitors in lung cancer

**DOI:** 10.20517/cdr.2019.85

**Published:** 2020-02-28

**Authors:** Barbara Rath, Adelina Plangger, Gerhard Hamilton

**Affiliations:** Department of Vascular Surgery, Medical University of Vienna, Vienna A-1090, Austria.

**Keywords:** Lung cancer, non-small cell lung cancer, small cell lung cancer, transformation, epidermal growth factor receptor, tyrosine kinase inhibitor, chemotherapy, drug resistance

## Abstract

Mutated or rearranged driver kinases in non-small cell lung cancer (NSCLC) cells are clinically amenable to treatment with tyrosine kinase inhibitors (TKIs) resulting in prolonged survival and significant benefit compared to cytotoxic chemotherapy. The most frequent genomic alterations are observed for epidermal growth factor receptor and anaplastic lymphoma kinase, which can be blocked by a range of specific TKIs in sequence. In clinics, resistance to TKIs emerges after approximately one year and comprises secondary mutations of the kinases (on-target) or alternative pathways circumventing the original kinase (off-target) alterations. A special feature of NSCLC is the occurrence of histological transformation to small cell lung cancer (SCLC) in up to 14% of cases, which, in general, is accompanied by resistance to the original TKIs. SCLC transformed tumors may be treated with the classical platinum/etoposide regimen but thus far there are no definitive guidelines. Four transformed pleural SCLC lines in our lab indicate the presence of a gradual NSCLC-SCLC shift with overlapping drug sensitivities. In conclusion, the treatment of NSCLC-SCLC transformed cancer cells would need a better chemosensitivity assessment using functional genomics to guide further therapy.

## Introduction

The tyrosine kinase inhibitors (TKIs) have revolutionized the treatment of lung cancer patients with respective mutations of so-called driver kinases and have demonstrated superior clinical activity compared to cytotoxic chemotherapy^[1]^. Furthermore, the inhibition of kinase signaling pathways are less toxic to normal tissues, resulting in considerably lower side effects^[2]^. Previously, chemotherapy using cisplatin in combination with etoposide, pemetrexed, or docetaxel was the standard of care for metastatic non-small cell lung cancer (NSCLC) and revealed five-year survival rates below 10%^[3]^. In the last decade, the proliferation of a part of the NSCLC cells was found to depend on a range of mutated or rearranged tyrosine kinases that could be targeted by specific inhibitors, which are preferentially directed to the ATP binding pockets of these enzymes. The most important altered kinases comprise epidermal growth factor receptor (EGFR) with a frequency of 15%-20%, HER2 with 2%, rearranged anaplastic lymphoma kinase (ALK) with 2%-7%, and rearranged ROS1 with a frequency of 1.7%^[1]^. Ligand binding to EGFR induces dimerization with other ERBB family members and the resulting receptor phosphorylation activates downstream RAS-RAF-MEK-ERK and RAS-PI3K-PTEN-AKT-mTOR pathways, leading to increased proliferation^[4]^. Although these mutations and rearrangements of EGFR, ALK, and ROS1 occur in a higher number of lung cancer cases, several other kinases show rare mutations and a wide range of different fusion genes and associated partners. Mutations result in constitutive activation of the kinases and rearrangements may activate kinases through the promoter function of the fusion partners. Historically, the EGFR TKIs erlotinib and gefitinib showed clinical activity against the tumors exhibiting corresponding mutations and a similar activity was proved for the TKI crizotinib and ALK-rearranged cancers^[1]^. NSCLCs become resistant to first-line EGFR-TKIs, within a median progression-free survival (PFS) of 9-13 months^[5]^. Recently, the use of frontline osimertinib, a third-generation TKI, has resulted in a PFS of 19 months and a response rate of approximately 80%^[6]^. In detail, pretreated T790M-mutant patients were reported to yield a median overall survival (OS) of 26.8 months and 12-, 24-, and 36-month survival rates of 80%, 55%, and 37% in response to osimertinib, respectively^[7]^. However, 20%-30% of EGFR mutated patients do not respond at all or only for a short time because of intrinsic resistance existing before treatment^[6]^. Furthermore, co-alterations in MET or other genes of the MAPK, PI3K, and Wnt/β-catenin signaling pathways and in cell cyle genes are linked to a poor response to EGFR-TKIs^[8]^.

**Figure 1:**
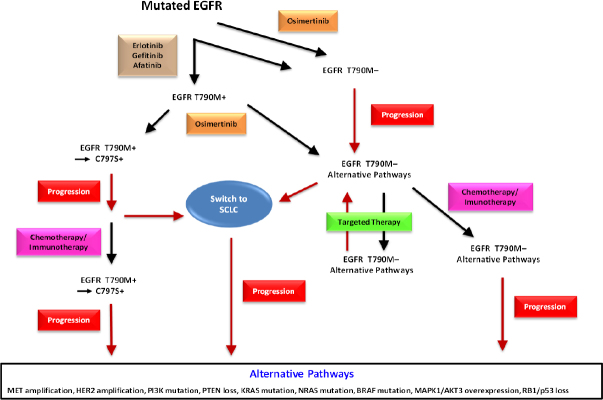
This scheme represents the sequence of therapeutic regimens in EGFR-mutated NSCLC. Tumors are treated with first-line agents erlotinib, gefitinib, or afatinib. Alternatively, the second-line osimertinib is applied directly or as actual second-line therapy. C797S may appear as further mutation of EGFR followed by progress and eventually by rescue chemotherapy. First-line osimertinib is rendered inactive by loss of T790M and the patients may be further treated by targeted agents against alternative pathway modulators and, upon progress, by rescue chemotherapy. Switch to a SCLC histotype may occur from both branches of therapy and result in cancer resistance and progress. SCLC: small cell lung cancer; EGFR: epidermal growth factor receptor; NSCLC: non-small cell lung cancer

Kinase inhibition exerts a strong pressure for the tumor cells to acquire resistance to TKIs through further kinase mutations or alternative pathways [Figure 1]. Therefore, despite the prominent anticancer activities of the TKIs, resistance develops invariably within approximately 12-18 months and the tumors relapse^[6]^. Further preclinical drug development provided access to second- and third-line TKIs that could be administered in sequence to inhibit the serially mutated kinases in order to prolong survival [Figure 1]. For example, erlotinib and gefinitib may be followed by afatinib or osimertinib for mutated EGFR and alectinib or brigatinib by lorlatinib or other ALK-directed agents. Crizotinib has been largely abandoned as a first-line agent due to its low intracranial activity. These agents are active against resistance caused by on-target mutations, i.e., further genetic modifications that alter the target structures of the respective kinases themselves. In contrast, non-target mutations result in activation or upregulation of other proliferation-stimulating pathways which bypass the dependence on the original mutated target and lead to tumor progress. Thus, the series of second- and third-line EGFR or ALK TKIs is eventually rendered inactive by activation of alternative kinases and, in the case of NSCLC, by a histological switch to a small cell lung cancer (SCLC) phenotype, which is not susceptible to the original TKIs^[9]^. Although formerly regarded to be rare with 1%-3% of cases, it became clear that NSCLC-SCLC transformation is more frequent with an incidence of up to 14% of cases and the resulting SCLC tumors exhibit varying characteristics and chemosensitivities^[9]^.

## Resistance to mutated EGFR-directed TKIs

EGFR-activating mutations are detected in approximately 15%-20% of NSCLC patients^[10]^. Accordingly, the available EGFR-directed TKIs are administered either in sequence or with the most active inhibitor, osimertinib, as first-line agent. However, tumor progress after the application of a sequence of TKIs is complex and not fully characterized. Repeat tissue or liquid biopsies during therapy have demonstrated multiple mechanisms of resistance, including secondary and tertiary EGFR mutations; bypass pathways such as c-MET or HER2 amplification; mutations of RAS, BRAF, PIK3CA, and other growth regulators; and novel fusion events and histological transformation^[8]^. The most common EGFR mutations are deletions in exon 19 (54%) and substitution of leucine with arginine at codon 858 (L858R, 41%), aside from a range of minor mutations^[11]^. Twenty percent of NSCLC patients with TKI resistance are related to *c-MET* gene amplification, which is not correlated with the presence of *T790M* mutation^[12]^. The *c-MET* gene amplification activates the ERBB3-PI3K signaling pathway directly*.* Patients sensitive to EGFR TKIs eventually progress after approximately 9-11 months compared to five months under chemotherapy. More than 60% of patients reveal resistance to first- or second-line inhibitors through a threonine-to-methionine substitution at the EGFR position 790 (T790M) in the ATP binding pocket of EGFR exon 20^[11,13]^. Metastatic EGFR T790M-positive NSCLC is treated with osimertinib, which is now approved for first-line treatment of EGFR-mutant lung cancer. Osimertinib binds irreversibly to the cysteine-797 residue at the ATP binding site of the EGFR but is subsequently rendered inactive by a further C797S mutation, against which several drugs are under development^[14]^. First-line application of osimertinib seems to result in different mechanisms of resistance, including a higher frequency of NSCLC-SCLC transformations. Resistance mechanisms involving *c-MET* and *HER2* amplification can be treated with crizotinib, which in addition to ALK also inhibits c-MET, and the HER2-targeting TKI afatinib, which blocks all types of ERBBs, respectively^[1,5,8]^. Signal transduction inhibitors and a host of novel agents interfering with alternative pathways are under preclinical and clinical development.

## NSCLC transformation to SCLC

When TKI resistance develops, patients generally undergo either a second tissue or a first liquid biopsy to determine the molecular mechanisms of refractoriness in order to guide the next treatment step and to predict the response^[13,15]^. Resistance to EGFR inhibitors through histological transformation of lung EGFR-mutant adenocarcinoma to SCLC has been reported in 3%-14% of cases in repeated biopsies series^[11,15-17]^. Typically, tumor tissues of lung adenocarcinoma patients with SCLC histology show expression of the typical markers synaptophysin, chromogranin, and CD56. In a recent study, 206 biopsies of EGFR TKI-resistant adenocarcinoma patients showed 21 SCLC transformed cases^[17]^. Patients experiencing SCLC transformation after third-generation EGFR TKI treatment (e.g., osimertinib) were included in the analysis. Many other isolated cases of histological transformation to SCLC under EGFR TKI treatment have been reported since 2006^[15,18,19]^. Biopsies from 71 patients with acquired resistance to osimertinib showed histological transformation in 14% overall and in 19% of samples after first-line osimertinib, in contrast to liquid biopsy-based results, which reported lower percentages of transformation^[20,21]^. NSCLC-SCLC transformation can be monitored in plasma samples by detection of increases of TP53 mutations and, more specifically, of copy number changes of SCLC-associated markers MYCL1, SOX2, and SOX4^[22]^. Accordingly, with 18% of cases, tertiary EGFR mutations were more common in patients treated with later-line osimertinib than first-line osimertinib with 6%^[22]^.

Transformation of NSCLC into SCLC may be due to a combined histology or a true switch of the histotype^[9]^. However, almost every transformed SCLC retained the original EGFR activating mutation of the parent adenocarcinoma and, correspondingly, strong and durable response to EGFR TKIs were found initially. These data are in favor of a true transformation and exclude a possible preexistent mixed histology^[19,23,24]^. The underlying mechanism of this histological NSCLC-SCLC transition is not clear. However, loss of RB1 was proven in a series of 11 cases of transformed EGFR mutant NSCLC specimens^[9,24,25]^. Upon complete inactivation of the tumor suppressor genes *RB1* and *TP53* at baseline, the clonal branching of SCLC cells from adenocarcinoma became visible before the TKI-start and the risk of SCLC transformation was increased > 40 times^[17]^. Time to transformation from the initial advanced NSCLC ranged from 2 to 60 months (median = 17.8 months) and from TKI-start between 1.3 and 53.4 months (median = 15.8 months)^[26]^. The frequent mutations in *TP53*, *RB1*, and *PIK3CA* are also typical of classic SCLC^[27]^. However, *RB1* and *TP53* loss seems to be necessary, but not sufficient^[16,17,28]^. This tumor suppressor inactivation is accompanied by decreased EGFR expression, despite the persistence of the EGFR activating mutation^[9,24]^. SCLC is thought to originate from neuroendocrine cells localized within the airways wall, while adenocarcinoma seems to be more related to alveolar type II cells^[9,29]^. However, the development of SCLC from alveolar type II cells was detected in mouse models^[30]^. Cellular plasticity between NSCLC and SCLC support the concept of a mutual origin from pluripotent alveolar cells^[9,19]^. The combination of EGFR TKI treatment and genetic and epigenetic modifications, such as *RB1* loss and *EGFR* down-regulation, could switch NSCLCs towards a SCLC histotype^[9]^. SCLC transformation from NSCLC has rarely been observed in EGFR-wild type lung cancers or during ALK-targeted therapy and programed cell death-1 (PD-1/L)-directed immunotherapy^[31]^. In addition to transformation to SCLC, histological switch to other NSCLC subtypes such as squamous cell carcinoma, large-cell neuroendocrine carcinoma, or sarcoma after TKI resistance have all been published^[32]^.

## Treatment of NSCLC-SCLC transformed tumors

NSCLC-SCLC transformation complicates the choice of an efficient therapeutic strategy. Initial reports indicated that the prognosis of transformed SCLC is poor and standard chemotherapy for primary SCLC appeared to be ineffective^[26,33]^. However, recent studies have reported favorable outcome after conventional systemic chemotherapy as applied for SCLC^[25,34,35]^. Platinum-etoposide was associated with an overall response rate (ORR) of 54% and a median PFS of 3.4 months^[26]^. Thus, patients with SCLC combined with NSCLC and SCLC transformed from adenocarcinoma should be offered standard therapies for SCLC^[26]^. Administration of immunotherapy with PD-1 or PD-L1 inhibitors for 17 patients with NSCLC-SCLC transformation showed no responders. Patients progressing on platinum-etoposide chemotherapy were treated with taxanes, yielding an ORR of 50% and a median PFS of 2.7 months. *In vitro*, osimertinib-resistant cells with SCLC transformation were more sensitive to paclitaxel compared with osimertinib-sensitive cells^[23,24]^. The median OS from diagnosis was 31.5 months, and median OS from transformation was 10.9 months. Following transformation to SCLC, central nervous system metastases were observed in 64% patients. In most cases (84%), the EGFR-mutant tumors maintain the same mutation after SCLC transformation but show additional loss or mutation in *TP53* and *RB1* genes, which are frequently seen in SCLC^[17]^. Several clinical cases of SCLC transformation after osimertinib treatment were reported to respond well to platinum-based doublet chemotherapy^[36]^*.* However, the short median PFS and OS after histological transformation indicate that the currently used therapeutic protocols are inefficient. The respective study by Marcoux *et al.*^[26]^ is corroborated by a series of 39 TKI-treated SCLC-transformed NSCLCs and a retrospective European cohort of 48 such patients in respect to response rate and OS^[32]^. Further research efforts are focused on trying to assess SCLC transformation by the use of non-invasive biomarkers such as serum pro-gastrin-releasing peptide, neuron-specific enolase, and liquid biopsy^[22,37]^.

## Phenotype of NSCLC-SCLC transformed tumor cells

**Figure 2:**
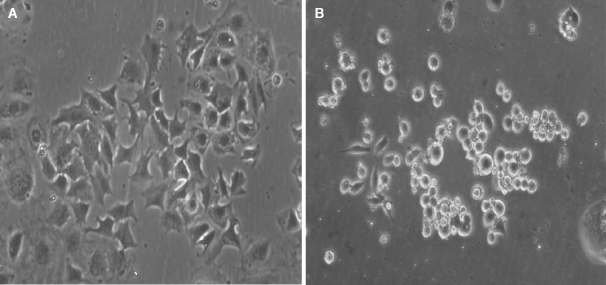
NSCLC-SCLC transformed pleural cell lines display either a phenotype still similar to a typical adenocarcinoma as in the case of BH15 (A) or a classical SCLC phenotype as in the case of BH20 (B). Both cell lines express SCLC markers. NSCLC: non-small cell lung cancer; SCLC: small cell lung cancer

Increased expression of neuroendocrine markers and decreased EGFR expression were detected in TKI resistant SCLC transformed cancers compared with resistant NSCLCs^[24]^. Usually, the cells change from an adherent widespread growth pattern to typical rounded suspension cultures under tissue conditions upon SCLC transformation [Figure 2]. Thus, following SCLC transformation, cancer cells became insensitive to EGFR-TKIs partly by downregulating the expression of EGFR protein and not by acquiring a secondary EGFR-mutation such as *T790M*^[9,19,38]^. However, 4 out of 19 cases retained the *T790M* mutation after transformation to SCLC^[17,25]^. Additionally, the RNA profile of SCLC-transformed NSCLC show a mixed transcriptome composed of classical SCLC features and a subset of mRNA more typically found in adenocarcinoma^[24]^. Tumor heterogeneity with concurrent patchy SCLC transformation and T790M EGFR resistance mutation in the same patients have been described^[39]^. For example, a patient who experienced resistance to therapy with afatinib through a histotype transformation to SCLC exhibited a locally confined chemosensitive switch of a part of the tumor^[9,40]^. Although EGFR mutations were identified in SCLC, these patients have had variable responses to EGFR inhibitors most likely related to the loss of EGFR expression at the protein level^[23,41,42]^. The transformed tumors are not always completely insensitive to EGFR-TKIs, as 52% of patients in the North American cohort received TKI therapy after transformation and a few cases showed clinical benefit from this treatment^[26]^. Subsequent re-challenge with the first-line TKIs has been tried as salvage treatment after progression of chemotherapy, yielding modest activity with a median PFS of 2.8 or 6.5 months following gefitinib or erlotinib treatment, respectively^[43]^.

## Conclusion

The most efficient therapy for SCLC-transformed NSCLC cells is not clear. The analysis of RNA expression of two NSCLC-SCLC transformed cell lines revealed a unique gene expression pattern in-between those of NSCLC and classical SCLC cells^[29]^. The morphologies of EGFR-mutant SCLCs are similar to those of typical SCLCs, but gene expression is different. These results may imply that EGFR-mutant SCLC has more phenotypic and functional plasticity than typical SCLC. The foundation treatment for SCLC is still chemotherapy but the response is far from satisfactory. In our lab, we have obtained four SCLC transformed cell lines from pleural effusions of NSCLC patients progressing after several TKI treatment periods. These cell lines exhibit a range of phenotypes resembling from adherent NSCLC with SCLC marker expression (BH15) to classical appearance of SCLC lines with small size cells in suspension culture (BH20). Whereas BH15 exhibits high resistance to cisplatin, BH20 is fully chemosensitive to the chemotherapeutics employed in therapy of SCLC, such as cisplatin, topotecan, and epirubicin (data not shown). However, for the BH20 SCLC line, tumorospheres were observed in tissue culture, which has been found to exhibit broad chemoresistance^[44]^. Surprisingly, the single approved drug for relapsed SCLC, topotecan, was not used in second-line treatment of advanced transformed SCLC, as well as the alternative epirubicin-based regimen but, instead, taxanes were administered. The example of BH15 demonstrates that tumors more similar to NSCLC after a partial change of the histotype may exhibit high resistance to cisplatin and are expected to respond poorly to the cisplatin/etoposide SCLC standard therapy. In such cases, second-line therapeutics such as topotecan or cyclosphosphamide/epirubicin/vincristine should be considered. Therapy of SCLC urgently needs new modalities of treatment such as inhibition of prostaglandin E synthase-1, which increases sensitivity to cisplatin and EGFR TKIs simultaneously^[45]^. However, functional testing of tumor cells may be essential to provide the best available chemotherapy for the NSCLC-SCLC transformed TKI-resistant tumors^[46]^.

## Declarations

### Copyright

© The Author(s) 2020.
